# Psychological Intelligence

**Published:** 1857-04-01

**Authors:** 


### NEW COMMISSIONER IN LUNACY.

It is our painful duty to announce the death of Dr. Hume, who for so long a period occupied the honourable post of one of the medical Commissioners in Lunacy. This physician was much honoured and esteemed by all who had the pleasure of his acquaintance. Dr. Nairne, one of the physicians to St. George's Hospital, has been appointed by the Lord Chancellor to the vacant commissionership.

---

### DR. CONSTANTINE SEIFERT.

This distinguished physician has recently been on a visit to England, as a Commissioner from the Russian Government, inspecting the public and private asylums of this country, and investigating the whole subject of lunacy. Previously to his visiting England, Dr. Seifert had been on a tour through Italy, France, and Belgium, on a similar errand. He visits the asylums of Germany before returning to Russia. Dr. Seifert is one of the physicians to the Imperial Lunatic Asylum of St. Petersburg. He is a man of great ability, knowledge, and intelligence. He leaves England, we are happy to record, highly impressed in favour of the English and Scotch asylums.

---

### DR. THOMAS MAYO.

The election of this eminent physician to the high and honourable office of *President of the Royal College of Physicians*, consequent upon the death of Dr. Paris, is entitled to more than a passing notice. We have, on more than one occasion, considered it to be our duty to oppose in this journal some of the medico-psychological and judicial opinions propounded by Dr. Mayo; we nevertheless are glad to avail ourselves of this opportunity of fully and freely acknowledging this physician's varied attainments, great talents, and undoubted private worth. As an accomplished and vigorous writer, acute and logical thinker, he occupies a high and enviable position among his contemporaries. Dr. Mayo's scholastic and literary acquirements are of the highest order. We feel assured that he will gracefully wear the laurels that have fallen upon him; and that all the anxious and responsible duties of his elevated position will be discharged with honour to himself and with satisfaction to the profession, of which he forms so bright an ornament.

